# Great insight created by tiny holes; celebrating 40 years of brain micropunch technique

**DOI:** 10.3389/fnana.2014.00061

**Published:** 2014-07-11

**Authors:** Denes V. Agoston

**Affiliations:** ^1^Anatomy, Physiology and Genetics, Uniformed Services UniversityBethesda, MD, USA; ^2^Experimental Traumatology, Department of Neuroscience, Karolinska InstituteStockholm, Sweden

**Keywords:** brain, miscrodissection, micropunch, mapping, biochemistry

Forty years ago, a two page long article (without references) was published in *Brain Research* by Palkovits (1973). The article's single figure show two coronal sections of the rat diencephalon with three tiny holes of thousand μm in diameter each; the first hole represented the removed paraventricular nucleus at the right side of the brain while the other two were of the habenular nuclei. The procedure—later named and officially known as brain micropunch method—helped to open a new chapter in neuroscience.

As is case for many great scientific advances, Miklós Palkovits' work occurred at the interfaces between two different disciplines. The first is classical neuroanatomy, a Hungarian “tradition” that started with Mihály Lenhossék, a contemporary and competitor of Santiago Ramón y Cajal who coined the name “astrocyte.” The tradition of excelling in neuroanatomy and neurohistology continued with János Szentágothai and Miklós Palkovits. The second discipline was neurochemistry. Improvements in analytical methods in the late 60s and early 70s increasingly enabled quantifying neurotransmitters, hormones, and metabolites from minute quantities of biological materials. Determining the concentrations of these and other molecules in the many functionally distinct brain regions promised a new level of understanding about complex biochemical and physiological processes of the central nervous system. Julius Axelrod pioneered work on how catecholamines, epinephrine, norepinephrine and dopamine—neurotransmitters of key neurological and psychiatric functions—are released and inactivated by reuptake. For this work, Julius Axelrod shared the Nobel Prize in Physiology or Medicine in 1970 with Bernard Katz and Ulf von Euler. It was Julius Axelrod who invited Miklós Palkovits to the National Institutes of Health (NIH) as “the guy who can dissect the suprachiasmatic nucleus” from the rat brain, the most important (if not the only) brain nucleus Julius Axelrod was interested in at the time.

Axelrod's invitation marked the beginning of an exceptionally fruitful collaboration between Miklós Palkovits and the Laboratory of Cell Biology (LCB) at the National Institute of Mental Health (NIH, Bethesda, Maryland). LCB was run by Michael Brownstein and it was a highly creative, intellectually stimulating and productive workplace. During his 6 month annual shifts at the NIH for ~30 years, Miklós Palkovits, in collaboration with neuroscientists and clinicians affiliated with various NIH laboratories, published dozens of papers in leading journals such as *Nature*, *Science*, and *PNAS* (to name a few). Miklós Palkovits and his collaborators have mapped neurochemical pathways of different transmitters, hormones, and metabolites, majorly contributing to the chemical anatomy of the brain. It is hopefully of some solace to young(er) scientists that the paper(s) describing the first topographic atlas of the catecholaminergic and cholinergic neurons in the rat brain (Jacobowitz and Palkovits, 1974; Palkovits and Jacobowitz, 1974) was barely accepted for publication. One reviewer stated, “I do not see that it adds a great deal to the existing literature.” It did and became one of Miklós Palkovits' citation classics in 1993.

Miklós Palkovits extended his collaborations to a worldwide network of colleagues and together they have extended mapping of neurotransmitters, neurochemicals, and hormones in various models of neurological disorders and in normal and diseased human brains. Working—and competing—with other giants of modern neuroscience like Tomas Hökfelt from the Swedish school of neuroscience, Miklós Palkovits also pioneered mapping of a new and exciting group of signaling molecules, the neuropeptides. He also established one of the first Human Brain Banks, a key repository of microdissected, micropunched brain regions for basic, translational, and applied research. The brain micropunch technique is now mainstream in the field of neuroscience, Leica Biosystems sells a Brain Punch Tissue Set for the “Palkovits Punch technique” and of course there is the “version 2” of his technique, the Laser Capture Microdissection System.

On a personal level, Miklós' knowledge and skills were instrumental for the development of a novel approach to identify some of the major molecular regulatory mechanisms in brain development. In my former laboratory at NIH, we set out to investigate how the expression of transmitter phenotypes in adult neurons is regulated during neurodifferentiation. Due to the vast phenotypic diversity of neurotransmitters across the brains of commonly used models (fly, worm, mammalian) used in developmental genetics, we would not have been able to identify the regulatory molecules we were searching for using homology cloning methods. Therefore, we developed an approach that involves sampling distinct brain regions at different stages of embryonic development, and probing the isolated nuclear proteins with DNA fragments (Dobi et al., 1997). Using this method we identified critical *cis*- as well as *trans*-regulatory elements involved in the developmental regulation of neuronal differentiation in the mammalian CNS (Dobi et al., 1995, 2000, 2006; Agoston et al., 1996, 2007; Dobi and Agoston, 1998; Agoston and Dobi, 2000; Szemes et al., 2006; Britanova et al., 2008; Gyorgy et al., 2008; Lee et al., 2008).

Our work began with dissecting hundreds and hundreds of embryonic rat brains beginning with as early as embryonic age day 12 brains. Miklós dissected the brains into phenotypically distinct regions up to postnatal day 28 and we isolated nuclear proteins from the isolated brain regions. Micropunching adult rodent brains without freezing can be challenging enough as is, but micropunching embryonic brains introduces a whole new array of issues due to size, high water content and texture. Because our micromethod for isolating nuclear proteins required fresh tissue, we asked Miklós to perform the micropunch technique on fresh embryonic brains as opposed to frozen brain slices, which he did. Then we performed thousands of DNA binding and footprinting assays, followed by molecular cloning, and so forth. In a way, this project closed the circle for Miklós by identifying some of the developmental regulators of neurotransmitters that he had started to map some 40 years ago.

Because Miklós likes numbers, I should state that Miklós Palkovits has co-authored 1000+ research papers, book chapters, abstracts, and proceedings. To be precise, as of December 09, 2013 (when the paper was submitted) he has authored and co-authored 663 abstracts, 59 book chapters, 8 books and monographies, co-edited 3 books, and published 722 peer-reviewed scientific publications in the most prestigious scientific journals. His papers have been citation classics and he has been nominated for the Nobel Prize twice. However, Miklós Palkovits has done so much more than “just” map the chemistry of the brain. His other (and equally important) work has been on connecting molecules and neuronal networks with complex neuronal functions. In addition to running the Human Brain Bank, Miklós has been also working on the first MRI atlas of cortical representation of pain, using his unparalleled anatomical knowledge, legendary organizational skills, and memory.

In the age of the Internet, Google, Wikipedia, and PubMed we tend to forget about the brilliance and hard work of scientists like Miklós Palkovits who with others collectively generated and cataloged a wealth of information before the digital age. Today, all information is only a click away… sort of. Ironically, while the literature is highly searchable, the scientific information is available in the same 19th–20th Century paper (or.pdf) format. There is still no electronic brain atlas that contains all of the biochemical, anatomical, and other knowledge he pioneered to collect (bar the Allen Brain Atlas, which currently contains mostly gene expression data). Hello, NIH! Hello, Google! Are you reading this?

Miklós, thank you for punching those little holes and contributing so much to modern neuroscience—and Happy (belated) 80th Birthday!

## Conflict of interest statement

The views, opinions, and/or findings contained in this article/presentation are those of the author and should not be interpreted as representing the official views or policies, either expressed or implied, of the Uniformed Services University. The author declares that the research was conducted in the absence of any commercial or financial relationships that could be construed as a potential conflict of interest.
